# Moral Foundations Predict Perceptions of Moral Permissibility of COVID-19 Public Health Guideline Violations in United States University Students

**DOI:** 10.3389/fpsyg.2021.795278

**Published:** 2022-02-02

**Authors:** Kathryn Bruchmann, Liya LaPierre

**Affiliations:** ^1^Department of Psychology, Santa Clara University, Santa Clara, CA, United States; ^2^Department of Mathematics, Seattle University, Seattle, WA, United States

**Keywords:** moral foundations, COVID-19, public health guideline adherence, prevention behaviors, moral judgments

## Abstract

In the United States, the COVID-19 pandemic has become highly politicized and highly moralized. The current study explored whether participants’ (*N* = 118) endorsements of binding (promoting group cohesion) versus individualizing (promoting care for individuals) moral foundations explained partisan differences in views and behaviors regarding COVID-19. Participants completed the Moral Foundations Questionnaire before they indicated how morally permissible they thought it was to violate COVID-19 mandates, report others’ violations, or not get vaccinated. Additionally, they indicated their own prevention behaviors. Results show that endorsement of both individualizing and binding foundations explain partisan differences in moral permissibility ratings. Political conservatism predicted greater endorsement of binding foundations which in turn predicted seeing COVID-19 violations and not getting vaccinated as more morally permissible, and predicted fewer self-reported prevention behaviors. Endorsement of individualizing foundations predicted seeing violations as less morally permissible and reporting others’ violations as more morally permissible.

## Moral Foundations Predicts Perceptions of COVID-19 Public Health Violations

During the COVID-19 pandemic, the decision not to follow public health recommendations can result in negative health consequences, and negative social consequences. For example, if a person chooses not to wear a mask indoors, social-distance from others, or get vaccinated, it could result in their family or friends choosing not to see them, or a business or venue not allowing them to enter. Additionally, these types of behaviors might elicit strong moral judgments from others. Indeed, the moralization of COVID-19 mitigation practices is thought by some to be responsible for the tensions between those who do adhere to practices and those who do not (Prosser et al., 2020). While around the world, strong national identification has been a predictor of adherence to COVID-19 guidelines (Van Bavel et al., 2021), in the United States specifically, political affiliation is one of the strongest predictors of adherence to mitigation practices (Deane et al., 2021); that is, Republicans have been less likely to follow mandates than Democrats. Indeed, political conservatives have reported being less concerned about the threat of getting COVID-19 (Malloy and Schwartz, 2020; Conway et al., 2021), and less concerned about the threat it might have to the United States population (Deane et al., 2021). Some research suggests that this is due to conservatives’ opposition to COVID-related restrictions, which makes them motivated to downplay the threat of COVID-19 (Conway et al., 2021). Republicans have been less supportive of government-mandated shutdowns, masking policies, and social-distancing compared with Democrats since the onset of the pandemic (Deane et al., 2021), and have been less likely to stay at home (Clinton et al., 2021) or report wearing masks (Howard, 2021). But why are attitudes about and compliance with COVID-19 restrictions so partisan?

One possible explanation is the endorsement of different morals. According to Moral Foundations Theory (e.g., Graham et al., 2009), liberals are more likely to endorse “individualizing” morals of *care for others* and *fairness or justice* than conservatives, whereas conservatives are more likely to endorse the “binding” morals of *loyalty to the ingroup*, *respect for authority* (particularly conservative authorities; Frimer et al., 2014), and *physical or spiritual purity* more than liberals. The individualizing morals focus on the treatment of individual people, while the binding morals are centered around group cohesiveness and duty (Graham et al., 2009). However, there is evidence that in the United States, conservatives often endorse both individualizing and binding foundations; that is, liberals show a larger gap between their endorsement of individualizing and binding foundations than conservatives do (Turner-Zwinkels et al., 2021). The five moral foundations have been shown to be stable across cultures (Dogruyol et al., 2019); however, a recent meta-analysis suggests that the relationship between politics and the endorsement of specific foundations may vary by context (Kivikangas et al., 2021).

Over the past decade, research has found that endorsement of moral foundations can explain political differences on a variety of issues, including support for stem cell research (Clifford and Jerit, 2013), attitudes toward the poor (Low and Wui, 2015), willingness to act on climate change (Dickinson et al., 2016), blaming victims versus perpetrators of violence (Niemi and Young, 2016; LaPierre and Bruchmann, 2021), and willingness to befriend political outgroup members (Bruchmann et al., 2018). Additionally, relevant to the COVID-19 pandemic, research has found a link between endorsement of the purity foundation with vaccine hesitancy (Amin et al., 2017; Karimi-Malekabadi et al., 2021); this is likely due to the belief that a vaccine would compromise physical purity.

### Moral Foundations and COVID-19

Since the onset of the COVID-19 pandemic, researchers have begun to focus on how moral foundations are related to COVID-19 related behaviors and moral transgressions. Ekici et al. (2021) found that people perceived moral transgressions as more permissible if they happened because of attempts to mitigate COVID-19 threats. For example, participants who endorsed the moral foundations of care, fairness, and purity were more likely to rate a target who missed a sibling’s wedding more favorably if they did so to minimize COVID-19 exposure than for another reason. Additionally, research has found that endorsement of care (Díaz and Cova, 2021) and fairness foundations (Chan, 2021) predicted more COVID-19 prevention behaviors. Some evidence suggests that this is because individualizing foundations predict a greater trust in science (Pagliaro et al., 2021). Across these articles, we see consistent evidence that endorsement of the individualizing foundations is important for COVID-19 behaviors and perceptions. But the question remains if moral foundations predict perceptions of *violations* of COVID-19 public health guidelines.

### The Present Research

The goal of the present research was to test whether endorsement of moral foundations would predict how permissible people thought it was to violate COVID-19 public health regulations and recommendations, and whether endorsement of moral foundations predicted actual COVID-19 prevention behaviors. Undergraduates completed the moral foundations questionnaire before rating the moral permissibility of behaviors violating COVID-19 guidelines, reporting others’ violations of these policies, and not receiving the COVID-19 vaccine. Additionally, participants rated their own prevention behaviors.

We predicted an asymmetrical mediational model such that endorsement of individualizing and binding foundations would both explain the partisan differences in perceptions of COVID-19 related behaviors, but in opposing ways. More specifically, we predicted that endorsement of individualizing foundations would be related to viewing violations of COVID-19 guidelines as less permissible, reporting others’ violations as more permissible, and not receiving the vaccine would be less permissible because violations of guidelines both could cause harm to others, and be seen as unfair to those that are adhering. Additionally, we expected that individualizing foundations, consistent with other work, would predict more prevention behaviors. However, we predicted that endorsement of binding foundations would be related to viewing violations of COVID-19 guidelines as more morally permissible. Specifically, due to conservatives’ belief in the moral importance of respecting conservative authorities (see Frimer et al., 2014 for examples), messaging from Republican authorities would likely play a large role in citizen’s attitudes and subsequent behaviors. In the United States specifically, President Donald Trump and other conservative leaders downplayed the threat of COVID-19 early on, often in opposition with the messages from the United States Chief Medical Advisor and infectious disease specialist Dr. Anthony Fauci and other public health experts (Durkee, 2021). We also predicted that binding foundations would be related to viewing reporting others’ violations as less morally permissible because it would violate loyalty to the ingroup, which would consist of other conservatives that are less likely to participate in COVID-19 prevention behaviors. Finally we predicted that binding foundations would be associated with viewing not receiving the vaccine as more permissible because endorsement of the purity foundation is associated with vaccine hesitancy (Amin et al., 2017).

## Methods

### Participants and Design

Undergraduates (*N* = 118) at a private Jesuit university in California participated in exchange for partial course credit. We made the *a priori* decision to recruit as many participants as possible during the school term. A *post hoc* Monte Carlo power analysis (Schoemann et al., 2017) suggests that we achieved between 55 and 62% power. Participants were on average 19.3 years old (*SD* = 0.90) and 60% self-categorized as women (35% men and 2% non-binary). Fewer than half self-categorized their race as white (39.2%), 39.2% self-categorized as Asian, 14.4% self-categorized as Hispanic or Latinx, and 7.2% self-categorized as other races. Participants’ political orientation skewed liberal with 58.4% identifying as Democrat, 14.4% as Independent, 7.2% as Republican, 2.4% as Libertarian, 1.6% as Green, 9.6% as other (mostly “not political”). Recruitment took place during spring 2021 when most courses were still online, and only a small number of first-year students were living in dorms. Only 17.8% were confirmed to have had COVID-19; of those, only one participant reported severe symptoms. The majority of the sample (75.4%) reported having a loved one who had been diagnosed with COVID-19, and 9.3% reported having lost a loved one to COVID-19. At the point of data collection, vaccinations were widely available; 82.2% reported already being vaccinated. This study was approved by the (Santa Clara University) IRB (ID: 20-11-1530), and all participants provided informed consent online before beginning the study.

### Materials and Procedures

First, participants completed the 30-item Moral Foundations Questionnaire (MFQ; Graham et al., 2009). In the first section of the MFQ, participants indicate how relevant statements such as “whether or not someone suffered emotionally” (*care*) are to their judgments of right and wrong (1 = *not at all relevant*, 6 = *extremely relevant*). In the second section of the MFQ, participants indicate their agreement with statements about each foundation (e.g., “people should be loyal to their family members, even when they have done something wrong”, *loyalty*; 1 = *completely disagree*, 6 = *completely agree*). Items were aggregated to form composites for individualizing foundations (α = 0.82), and binding foundations (α = 0.86).

Next, participants completed the 7-item Fear of COVID-19 scale (FCV-19; Ahorsu et al., 2020; α = 0.89 in the present sample). Participants indicated their agreement with items such as “When I watch news and stories about COVID-19 on social media, I become nervous or anxious” (1 = *strongly disagree*, 5 = *strongly agree*).

#### Permissibility of Behaviors

Participants then rated how morally permissible a series of 15 COVID-19 related behaviors were, independent of local, state, or federal guidelines. Participants were asked to assume all parties involved in the scenarios were not vaccinated. Behaviors were categorized as “major violations” (e.g., spending time with people after knowingly testing positive for COVID-19; α = 0.84), “minor violations” (e.g., playing contact sports without masks; α = 0.87), or “reporting violations” (e.g., telling authorities when someone does not comply with COVID-19 mandates; α = 0.69). One item also assessed how morally permissible it would be to not receive the COVID-19 vaccine after becoming eligible. Participants rated each of these behaviors on their moral permissibility (1 = *not at all morally permissible*, 6 = *totally morally permissible*).

Next, participants rated their compliance with specific COVID-19 related behaviors: how often they wash their hands, how often they maintain social-distance with others in public or how often they wear masks in public (1 = *never*, 5 = *frequently*; α = 0.601)^1^. And, participants rated their agreement with statements about their behaviors being in total compliance with local and state mandates, and CDC recommendations regarding COVID-19 (1 = *strongly disagree*, 6 = *strongly agree;*α = 0.98).

Finally, participants indicated their demographic information, including their political ideology (1 = *extremely liberal*, 6 = *extremely conservative*) before being probed for suspicion and debriefed.

## Results

### Preliminary Analyses

See Table 1 for descriptive statistics of all variables. From this table, we see that our participants reported exhibiting COVID-19 prevention behaviors; as a group, they reported social-distancing, masking, and handwashing, *t*(117) = 24.75, *p* < 0.001, *d* = 2.28, far above the midpoint of the scale (3). Similarly, participants reported their compliance with local, state, and federal COVID-19 guidelines to be above the midpoint of the scale (3.5), *t*(117) = 10.09, *p* < 0.001, *d* = 0.93. Consistent with previous research (Atari et al., 2020), women (*M* = 4.85, *SD* = 0.54) were more likely to endorse individualizing foundations than men [*M* = 4.48, *SD* = 0.72; *t*(111) = 3.14, *p* = 0.002, *d* = 0.62]; additionally, as seen in other research (e.g., Capraro and Barcelo, 2020; Galasso et al., 2020), women (*M* = 4.37, *SD* = 0.49) reported more prevention behaviors than men [*M* = 4.12, *SD* = 0.65; *t*(111) = 2.24, *p* = 0.027, *d* = 0.56].

**TABLE 1:** Descriptive statistics for all study variables.

Measures	Possible	Mean	SD
1. Political ideology	1–6	2.21	0.98
2. Individualizing foundations	1–7	4.73	0.64
3. Binding foundations	1–7	3.34	0.73
4. Fear of COVID-19	1–5	2.27	0.94
5. Permissibility of major violations	1–6	2.11	0.99
6. Permissibility of minor violations	1–6	3.10	1.18
7. Permissibility of reporting	1–6	4.16	1.29
8. Permissibility of not receiving vaccine	1–6	2.50	1.44
9. Prevention behaviors	1–5	4.27	0.56
10. Compliance	1–6	4.68	1.27

### Do Moral Foundations Explain Political Differences?

**FIGURE 1:**
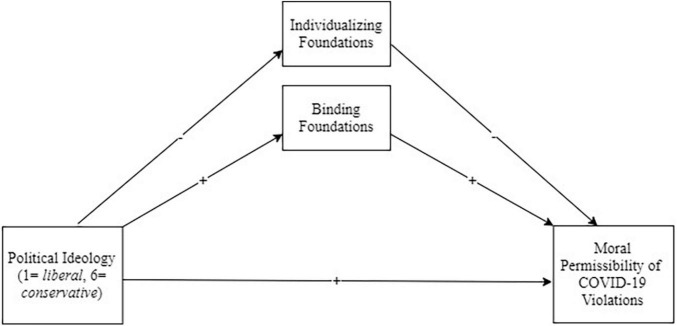
Mediation model: Political identify (IV), moral foundations (Mediators), moral permissibility (DVs).

In order to test whether moral foundations explain political differences in perceptions of the moral permissibility of COVID-19 guideline violations and prevention behaviors, we conducted mediation analyses using PROCESS (Hayes, 2017) model 4 with continuous political ideology as the predictor (x), our outcomes (ratings of moral permissibility of major COVID-19 violations, minor violations, reporting others’ violations, not getting vaccinated, and actual compliance behaviors) as the dependent measures (y), Individualizing and Binding foundations as the mediators (m), and FCV-19 as a covariate^2^. See Figure 1 for the predicted model. All analyses used 5,000 bootstrap samples.

For all outcomes, the *a* paths to binding foundations from political ideology were significant, β = 0.30, SE = 0.06, *t* = 4.88, *p* < 0.001, 95% CI (0.18, 0.43). The more conservative participants were, the more they endorsed binding foundations, consistent with previous research. The *a* paths to individualizing foundations from political ideology were also significant, β = −0.16, SE = 0.06, *t* = −2.67, *p* = 0.009, 95% CI (−0.28, −0.04); the more conservative participants were, the less they endorsed individualizing foundations.

#### Major Violations

The overall model was significant for major violations, *R*^2^ = 0.09, *F*(4,112) = 2.72, *p* = 0.033. The *b* path from binding foundations to major violations was significant, β = 0.41, SE = 0.15, *t* = 2.73, *p* = 0.007, 95% CI (0.11, 0.70), as was the *b* path from individualizing foundations, β = −0.37, SE = 0.15, *t* = −2.38, *p* = 0.019, 95% CI (−0.67, −0.06). The direct effect of political ideology on the moral permissibility of major violations (*c* path) was not significant; however, the indirect effect was, β = 0.18, SE = 0.07, 95% CI (0.05, 0.34). The mediation went through both binding, β = 0.12, SE = 0.07, 95% CI (0.01, 0.28), and individualizing foundations β = 0.06, SE = 0.04, 95% CI (0.00, 0.14). In other words, the more participants identified as liberal, the more they endorsed individualizing foundations, and, in turn, the more morally permissible they viewed reporting other people’s COVID-19 guideline violations to be.

#### Minor Violations

The overall model was significant for minor violations, *R*^2^ = 0.17, *F*(4,112) = 5.84, *p* < 0.001. The *b* path from binding foundations to the moral permissibility of minor violations was significant, β = 0.46, SE = 0.17, *t* = 2.72, *p* = 0.008, 95% CI (0.12, 0.79), but the *b* path from individualizing foundations was not, β = −0.23, SE = 0.17, *t* = −1.33, *p* = 0.187, 95% CI (−0.58, 0.11). Additionally, there was an effect of the covariate FCV-19 on minor violations, β = −0.42, SE = 0.11, *t* = −3.86, *p* < 0.001, 95% CI (−0.64, −0.21), such that a greater FCV-19 was associated with viewing minor violations as less morally permissible. The direct effect of political ideology on minor violations (*c* path) was not significant, β = −0.28, SE = 0.12, *t* = −0.22, *p* = 0.824, 95% CI (−0.27, 0.21), however, the indirect effect was, β = 0.18, SE = 0.08, 95% CI (0.04, 0.34). The mediation went through binding, β = 0.04, SE = 0.03, 95% CI (−0.01, 0.12), but not individualizing foundations, β = 0.14, SE = 0.07, 95% CI (−0.01, 0.12). In other words, the more conservative participants were, the more they endorsed binding foundations, and the more morally permissible they found minor COVID-19 guideline violations to be.

#### Reporting Others’ Violations

The overall model was significant for reporting others’ violations, *R*^2^ = 0.18, *F*(4,114) = 12.57, *p* < 0.001. The *b* path from binding foundations to the moral permissibility of reporting others’ violations was not significant, β = −0.26, SE = 0.18, *t* = −1.47, *p* = 0.144, 95% CI (−0.62, 0.09), but the *b* path from individualizing foundations was, β = 0.63, SE = 0.19, *t* = 3.38, *p* = 0.001, 95% CI (0.26, 1.00). Additionally, there was an effect of the covariate FCV-19 on reporting, β = 0.38, SE = 0.12, *t* = 3.31, *p* = 0.001, 95% CI (0.15, 0.62), such that a greater fear of FCV-19 was associated with viewing reporting others’ violations as more permissible. The direct effect of political ideology on reporting violations (*c* path) was not significant, β = −0.14, SE = 0.13, *t* = −1.09, *p* = 0.278, 95% CI (−0.40, 0.11), nor was the overall indirect effect, β = −0.18, SE = 0.09, 95% CI (−0.36, 0.00). However, there was significant mediation through individualizing foundations, β = −0.10, SE = 0.06, 95% CI (−0.23, −0.02). In other words, the more liberal participants were, the more they endorsed individualizing foundations, and the more morally permissible they viewed reporting other people’s COVID-19 guideline violations to be.

#### Vaccines

The overall model was significant for moral permissibility of not receiving the COVID-19 vaccine, *R*^2^ = 0.09, *F*(4,112) = 2.63, *p* = 0.038. The *b* path from binding foundations to the permissibility of not being vaccinated was significant, β = 0.48, SE = 0.16, *t* = 2.18, *p* = 0.032, 95% CI (0.04, 0.91), as was the *b* path from individualizing foundations, β = −0.48, SE = 0.23, *t* = −2.12, *p* = 0.038, 95% CI (−0.93, −0.03). The direct effect of political ideology on permissibility of not getting vaccinated (*c* path) was not significant, β = 0.01, SE = 0.16, *t* = 0.05, *p* = 0.961, 95% CI (−0.31, 0.33); however, the overall indirect effect was, β = 0.22, SE = 0.09, 95% CI (0.04, 0.42). There was significant mediation through individualizing, β = 0.08, SE = 0.05, 95% CI (0.00, 0.18), but not binding foundations (despite the significant direct effect), β = 0.15, SE = 0.08, 95% CI (−0.00, 0.32). In other words, the more liberal the participants were, the more they endorsed individualizing foundations, and thus, the less morally permissible they thought it was for people to choose not to get vaccinated.

#### Prevention Behaviors and Compliance With Guidelines

The overall model was significant for prevention behaviors, *R*^2^ = 0.14, *F*(4,112) = 4.46, *p* = 0.002. The *b* path from binding foundations to prevention behaviors was significant, β = −0.21, SE = 0.08, *t* = 1.29, *p* = 0.01, 95% CI (−0.38, −0.05), but the *b* path from individualizing foundations was not, β = 0.11, SE = 0.09, *t* = 1.29, *p* = 0.201, 95% CI (−0.06, 0.28). Additionally, there was an effect of the covariate FCV-19 on prevention behaviors, β = 0.15, SE = 0.05, *t* = 2.78, *p* = 0.007, 95% CI (0.04, 0.25), such that a greater FCV-19 was associated with exhibiting more prevention behaviors. The direct effect of political ideology (*c* path) on prevention behaviors was not significant, β = −0.01, SE = 0.06, *t* = −0.25, *p* = 0.808, 95% CI (−0.13, 0.10), however, the indirect effect was, β = −0.08, SE = 0.03, 95% CI (−0.16, −0.02). The mediation went through binding, β = −0.07, SE = 0.03, 95% CI (−0.13, −0.01), but not individualizing, β = −0.02, SE = 0.02, 95% CI (−0.06, 0.01). In other words, the more politically conservative participants were, the more they endorsed binding foundations, and the less likely they were to report engaging in COVID-19 prevention behaviors.

For self-reported compliance with COVID-19 guidelines, the model was non-significant, *R*^2^ = 0.05, *F*(4,112) = 1.54, *p* = 0.221.

## Discussion

This study demonstrates that moral foundations are important to consider when examining attitudes and behaviors during the COVID-19 pandemic. As predicted, both endorsement of binding foundations and individualizing foundations mattered for perceptions of the moral permissibility of COVID-19 related behaviors. Specifically, we saw evidence that higher endorsement of individualizing foundations was associated with viewing major violations of COVID-19 regulations as less morally permissible; likely because major violations of COVID-19 guidelines, such as spending time with someone after knowingly testing positive for the illness, can be seen as both causing harm to others and as unjust or unfair for those who are adhering to guidelines. Additionally, we saw evidence that higher endorsement of the binding foundations was associated with viewing both major and minor violations of COVID-19 regulations as more morally permissible. Research suggests that conservatives—despite being more likely to endorse the authority foundation— view obedience as more positive when it is toward conservative or in-group authorities (Frimer et al., 2014); throughout the COVID-19 pandemic, experts in the United States such as the Center for Disease Control have often been directly at odds with conservative leadership which might undermine their authority.

Inconsistent with previous research (Chan, 2021; Ekici et al., 2021; Pagliaro et al., 2021), we did not see that individualizing foundations predicted more self-reported COVID-19 prevention behaviors, but we did see that binding foundations were related to reporting fewer prevention behaviors such as hand-washing, masking, and social-distancing. This is contrary to recent research in a French population that found that endorsing the binding foundations of authority and purity were associated with *increased* prevention behaviors (Díaz and Cova, 2021), suggesting that the effects we found may be unique to the U.S. American population. Indeed, the COVID-19 pandemic is more politicized and polarized in the United States than other countries (Mordecai and Connaughton, 2020). Despite this, we saw overall that our sample reported very high levels of prevention behaviors, which could be due to social desirability; testing behaviors more objectively would perhaps provide greater variability in results.

Those who endorsed individualizing foundations less were also more likely to view reporting violations as morally permissible. This finding is interesting when considering that generally individualizing foundations are associated with less punitiveness (Silver and Silver, 2017). However, since regulations are in place in order to prevent harm and protect those who are more vulnerable, it may be that endorsing the care foundation makes reporting COVID-19 violations more acceptable. Indeed Ekici et al. (2021) found that moral violations were seen as more permissible when people were exhibiting them to avoid the spread of COVID-19.

Finally, we saw that higher endorsement of individualizing foundations was linked to viewing not getting vaccinated against COVID-19 as *less* morally permissible, and endorsement of binding foundations was linked to viewing not getting vaccinated as *more* morally permissible. Because vaccinations are designed not just to protect the self but to protect the public from transmission, it is logical that endorsement of the care/harm foundation would be related to seeing vaccination as a moral obligation. Similarly because of the disparities in access (e.g., Joseph and Dore, 2021) to vaccinations worldwide, not getting vaccinated when able may be seen as unjust. However, endorsement of the binding foundation of purity has also been linked to vaccine hesitancy (Amin et al., 2017), which can explain why others’ hesitancy might be seen as more permissible.

### Limitations

This study was not without limitations. First and foremost, our sample was small, and homogeneous in terms of age (young), politics (liberally skewed), and gender (predominantly female). All three of these factors could influence our results given that age is one of the strongest predictors of risk and severity of COVID-19 infections (Hu et al., 2021), political liberalism is associated with both perceptions of COVID-19 (Conway et al., 2021) and moral foundations (e.g., Graham et al., 2009), and that women are more likely to endorse individualizing foundations than men (Atari et al., 2020), and follow COVID-19 prevention behavior guidelines than men (e.g., Galasso et al., 2020). Future research should test these effects in a sample with more demographic variability in order to replicate or extend these findings. Additionally, the timing of our study may have influenced results; vaccines were already available to our participants (and, most were vaccinated), but the delta variant and other waves of cases had not arrived yet, so participants may have been thinking more retrospectively. Testing these effects during outbreaks of new strains to test how current case rates influence people’s perceptions of moral permissibility would help to paint a full picture.

Additionally, future research should include a measure of participants’ endorsement of the moral values of liberty, which has become recognized as a sixth moral foundation in recent years (Iyer et al., 2012). Mentions of violations of personal freedoms are rampant among conservative politicians (e.g., Perry et al., 2020) who are against masking laws and other COVID-19 mandates, and personal endorsement of liberty may be a strong predictor of how COVID-19 violations are viewed, and particularly whether people view it as morally permissible to not receive the COVID-19 vaccine (e.g., Amin et al., 2017). And finally, future research should consider other factors such as religiosity (e.g., Malka et al., 2012), (mis)trust in science and/or medicine (e.g., Pagliaro et al., 2021) as tests of alternate mechanisms of the political differences in perceptions of COVID-19 related behaviors.

### Conclusion

The political polarization of the COVID-19 pandemic in the United States can be further understood by considering the role of moral foundations. The present study is important both in understanding the far-reaching implications of Moral Foundations Theory, but is also important in understanding what contributes to whether or not people follow COVID-19 guidelines, and how people who do not follow guidelines are viewed. As policies continue to be informed by social science (see Van Bavel et al., 2020), understanding what makes people view violations as morally permissible or not can help public health officials generate targeted campaigns to liberals versus conservatives to be more effective in curbing the spread of COVID-19.

## Data Availability Statement

The raw data supporting the conclusions of this article will be made available by the authors, without undue reservation.

## Ethics Statement

The studies involving human participants were reviewed and approved by the Santa Clara University IRB. The participants provided their written informed consent to participate in this study.

## Author Contributions

KB and LL contributed to the formulation of the research question and edited and revised the manuscript. KB completed the analyses and wrote the initial draft of the manuscript. Both authors contributed to the article and approved the submitted version.

## Conflict of Interest

The authors declare that the research was conducted in the absence of any commercial or financial relationships that could be construed as a potential conflict of interest.

## Publisher’s Note

All claims expressed in this article are solely those of the authors and do not necessarily represent those of their affiliated organizations, or those of the publisher, the editors and the reviewers. Any product that may be evaluated in this article, or claim that may be made by its manufacturer, is not guaranteed or endorsed by the publisher.

## Footnotes

1 This low value is likely due to low variability in self-reported masking, perhaps because this study took place in California with strict public masking laws.

2 When including gender as an additional covariate, all patterns remained the same; however, the mediation through Individualizing foundations dropped to marginal significance for the Major Violations outcome.
